# Large oncosomes contain distinct protein cargo and represent a separate functional class of tumor-derived extracellular vesicles

**DOI:** 10.18632/oncotarget.3598

**Published:** 2015-03-14

**Authors:** Valentina R. Minciacchi, Sungyong You, Cristiana Spinelli, Samantha Morley, Mandana Zandian, Paul-Joseph Aspuria, Lorenzo Cavallini, Chiara Ciardiello, Mariana Reis Sobreiro, Matteo Morello, Geetanjali Kharmate, Su Chul Jang, Dae-Kyum Kim, Elham Hosseini-Beheshti, Emma Tomlinson Guns, Martin Gleave, Yong Song Gho, Suresh Mathivanan, Wei Yang, Michael R. Freeman, Dolores Di Vizio

**Affiliations:** ^1^ Division of Cancer Biology and Therapeutics, Departments of Surgery, Biomedical Sciences and Pathology and Laboratory Medicine, Samuel Oschin Comprehensive Cancer Institute, Cedars-Sinai Medical Center, Los Angeles, CA, USA; ^2^ The Urological Diseases Research Center, Boston Children's Hospital, Boston, MA, Department of Surgery, Harvard Medical School, Boston, MA, USA; ^3^ Women's Cancer Program, Cedars-Sinai Medical Center, Los Angeles, CA, USA; ^4^ Department of Experimental and Clinical Biomedical Science, University of Florence, Florence, Italy; ^5^ Experimental Pharmacology Unit, Department of Research, IRCCS-Istituto Nazionale Tumori G. Pascale, Naples, Italy; ^6^ Vancouver Prostate Centre, Department of Urologic Sciences, University of British Columbia, BC, Canada; ^7^ Department of Life Sciences, Pohang University of Science and Technology, Pohang, Republic of Korea; ^8^ Department of Biochemistry, La Trobe Institute for Molecular Science, La Trobe University, Bundoora, Australia

**Keywords:** Extracellular Vesicles, SILAC Proteomics, Cancer metabolism, Tumor progression, Amoeboid blebbing

## Abstract

Large oncosomes (LO) are atypically large (1-10μm diameter) cancer-derived extracellular vesicles (EVs), originating from the shedding of membrane blebs and associated with advanced disease. We report that 25% of the proteins, identified by a quantitative proteomics analysis, are differentially represented in large and nano-sized EVs from prostate cancer cells. Proteins enriched in large EVs included enzymes involved in glucose, glutamine and amino acid metabolism, all metabolic processes relevant to cancer. Glutamine metabolism was altered in cancer cells exposed to large EVs, an effect that was not observed upon treatment with exosomes. Large EVs exhibited discrete buoyant densities in iodixanol (OptiPrep^TM^) gradients. Fluorescent microscopy of large EVs revealed an appearance consistent with LO morphology, indicating that these structures can be categorized as LO. Among the proteins enriched in LO, cytokeratin 18 (CK18) was one of the most abundant (within the top 5^th^ percentile) and was used to develop an assay to detect LO in the circulation and tissues of mice and patients with prostate cancer. These observations indicate that LO represent a discrete EV type that may play a distinct role in tumor progression and that may be a source of cancer-specific markers.

## INTRODUCTION

Extracellular vesicles (EVs) that originate from cancer cells are gradually and consistently emerging as important regulators of tumor progression [1]. Nano-sized particles called exosomes (~100 nm diameter) are generally considered to originate from multivesicular bodies (MVB) of the late endocytic pathway [2]. In addition to exosomes, tumor cells also produce larger EVs (500 to >1000 nm diameter), referred to in general terms as microvesicles (MVs) [3-5] or ectosomes. MVs seem to be generated by budding of the plasma membrane. Exosomes and other EVs play important functions dictated by their cell of origin and their content [6]. After years of research aimed to identify unequivocal exosome markers, it is now clear that “universal exosome markers” are difficult to find, and recent proteomic studies suggest that exosome populations are diverse and can be enriched with distinct proteins that impart specific functions to the particles [7]. Similarly, it is not known whether specific proteins can be expressed in exosomes versus plasma membrane-derived EVs, such as ectosomes/MVs, and vice-versa.

We recently demonstrated the existence of an atypically large class of EVs (1-10 μm in diameter), termed large oncosomes (LO), which can be byproducts of non-apoptotic plasma membrane blebbing induced by silencing of the cytoskeletal regulator Diaphanous-related formin-3 (DIAPH3), by overexpression of oncoproteins such as MyrAkt1, HB-EGF, and caveolin-1 (Cav-1), or by activation of the EGFR [8-11]. In comparison to exosomes and MVs, LO are still a poorly characterized EV type [12]. Using expression of Cav-1, a circulating marker of metastatic prostate cancer [13] to quantify LO, we demonstrated a significant increase of LO enumeration in the circulation of both TRAMP mice, which harbor rapidly progressing prostate tumors, and patients with metastatic disease [9]. However, because Cav-1 is also detected in exosomes, and our earlier method for LO discrimination was simply based on particle size being >1 μm, we went on and analyzed the protein content of LO and nano-sized EVs using mass spectrometry to identify specific cargo.

Here we report a quantitative duplex stable isotope labeling of amino acids in cell culture (SILAC) analysis of large and nano-sized EVs isolated by differential centrifugation from DU145 cells silenced for DIAPH3. We identified uniquely expressed and differentially expressed proteins in the two preparations. Metabolism emerged as a biological process enriched in large versus nano-sized EVs, and this result was functionally validated. In order to validate the mass spectrometry data, to separate EVs from particulate material, and to determine the density of large EVs, we applied equilibrium centrifugation in OptiPrep^TM^ gradients using an upward displacement modality [14-17]. Among the proteins enriched in large EVs, cytokeratin 18 (CK18) emerged as a potential marker for tumor-derived EVs in tissues and in plasma. Our findings suggest that LO, compared to exosomes, contain different protein cargo, are functionally distinct, and harbor proteins that might be employed in their functional characterization and used as tissue and circulating markers of disease progression.

## RESULTS

### Silencing of DIAPH3 results in increased shedding of large EVs

**Figure 1:**
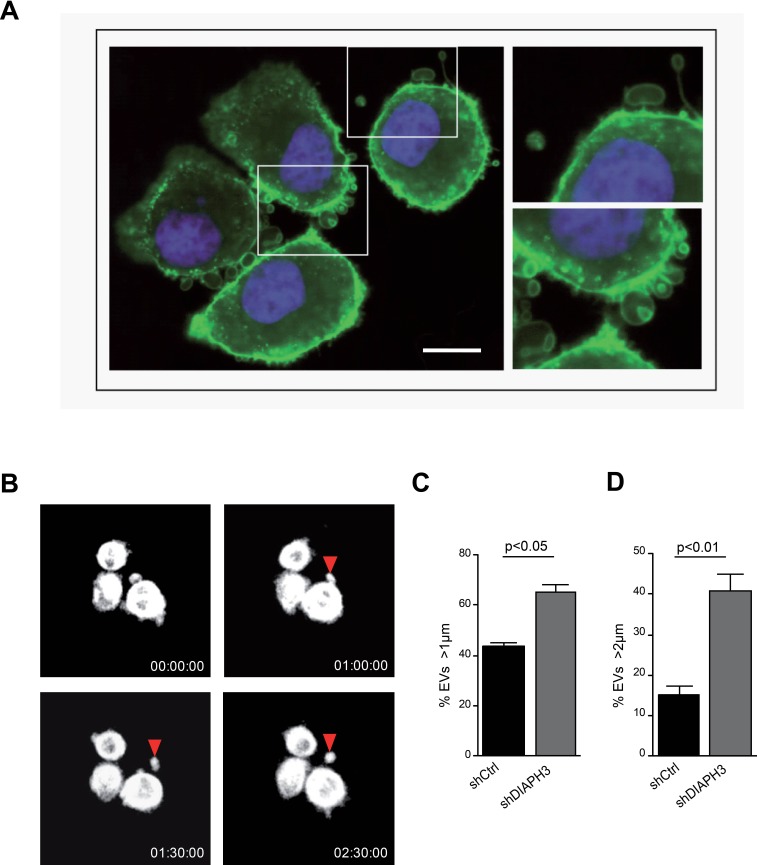
Silencing of DIAPH3 results in increased shedding of large EVs (A) Membrane blebbing and shedding of EVs of variable size (insets) from DU145 cells stably expressing DIAPH3 shRNA, stained with CTxB-FITC (X 63). Scale bar, 20 μm. (B) 30 min-1h interval frames (from Supplementary Movie 1), acquired by real-time confocal microscopy of DIAPH3-silenced DU145 over-expressing RFP-tubulin. The arrow points to a membrane bleb that is released as a large oncosome. (C, D) EVs from DIAPH3-silenced or parental DU145 cells were analyzed by flow cytometry.

Consistent with published results, silencing of the cytoskeletal regulator DIAPH3 (shDIAPH3) in DU145 cells resulted in the formation of large plasma membrane blebs (1-10 μm in diameter) (Figure 1A, Supplementary Movie 1) [8, 10], which were shed into the medium as large EVs (Figure 1B). We observed a higher amount of total protein in large EVs from shDIAPH3 than control cells, collected from the same number of donor cells by differential centrifugation (10,000 × g) [3, 10, 18] (Supplementary Figure 1A). Flow-cytometry (FACS) analysis with specific size beads (1-10 μm), previously employed by us to quantify EVs in the size range of LO [10], revealed a significantly higher number of EVs > 1 μm in media from DIAPH3 silenced versus control cells (Figure 1C, Supplementary Figure 1B). The result was even more robust when we gated for events larger than 2 μm (Figure 1D), consistent with our previous observation that the modal distribution of LO shed from prostate cancer cells is 2-3 μm [10].

### Mass spectrometry identifies differentially expressed and unique proteins in large and nano-sized EVs

Nanoparticle Tracking Analysis (NTA) showed that shDIAPH3 DU145 cells also produce higher numbers of nano-sized EVs [11]; consequently, we used this system as a model to quantitatively compare the protein composition of large and nano-sized EVs, isolated by differential centrifugation, by SILAC. To minimize false-positive hits, two independent SILAC label-swap experiments were conducted. Differentially expressed proteins were identified by an integrative statistical hypothesis testing method with log2-scaled and quantile normalized SILAC intensities [19-21].

**Figure 2:**
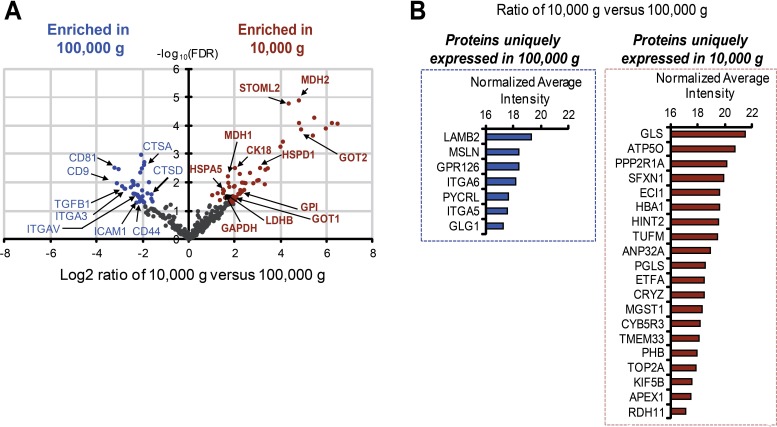
Identification of unique and differentially expressed proteins in large and nano-sized EVs (A) Volcano plots of the log2 ratio of the averaged, normalized SILAC intensities against the FDR of the differential expression between large and nano-sized EVs. Red and blue dots correspond to proteins enriched in large EVs and nano-sized EVs, respectively. (B) Bar plots show the abundance of unique proteins quantified in large (right) and nano-sized EVs (left). The horizontal axis represents the normalized average log2 ion intensities of uniquely identified proteins.

A total of 407 proteins were identified, among which 103 (approximately 25% of the total) were differentially represented in large and nano-sized EVs, with a false discovery rate (FDR) < 0.05, and fold change ≥ 2 (Figure 2A, and Supplementary Figure 2A, B). A volcano plot of the differentially expressed proteins showed a noticeable enrichment in nano-sized EVs for tetraspanin family members CD81 and CD9 (Figure 2A, Supplementary Figure 2B), proteins known to be over-represented in exosomes [22-24]. Additional top-ranked differentially expressed proteins (>4 fold) in nano-sized EVs included cell adhesion molecules, such as integrin-α3 (ITGA3), integrin-αV (ITGAV), intercellular adhesion molecule (ICAM) and CD44. Transforming growth factor-β1 (TGFβ1), which has been linked to cancer progression for its role in the tumor microenvironment [25, 26], and which participates in exosome-mediated biological functions [27, 28], was 6-fold more abundant in nano-sized versus large EVs (Figure 2A). Cathepsin proteases (CTSD and CTSA), usually associated with the plasma membrane or secreted into the extracellular environment [29], were also enriched in nano-sized EVs.

In contrast, proteins enriched in large EVs included glyceraldehyde 3-phosphate dehydrogenase (GAPDH), glucose phosphate isomerase (GPI), lactate dehydrogenase B (LDHB), heat shock 70kDa protein 5 (HSPA5), malate dehydrogenase (MDH) and aspartate transaminase (GOT) family members (Figure 2A, Supplementary Figure 2A). These proteins are all involved in metabolic processes relevant to cancer [30, 31].

**Figure 3:**
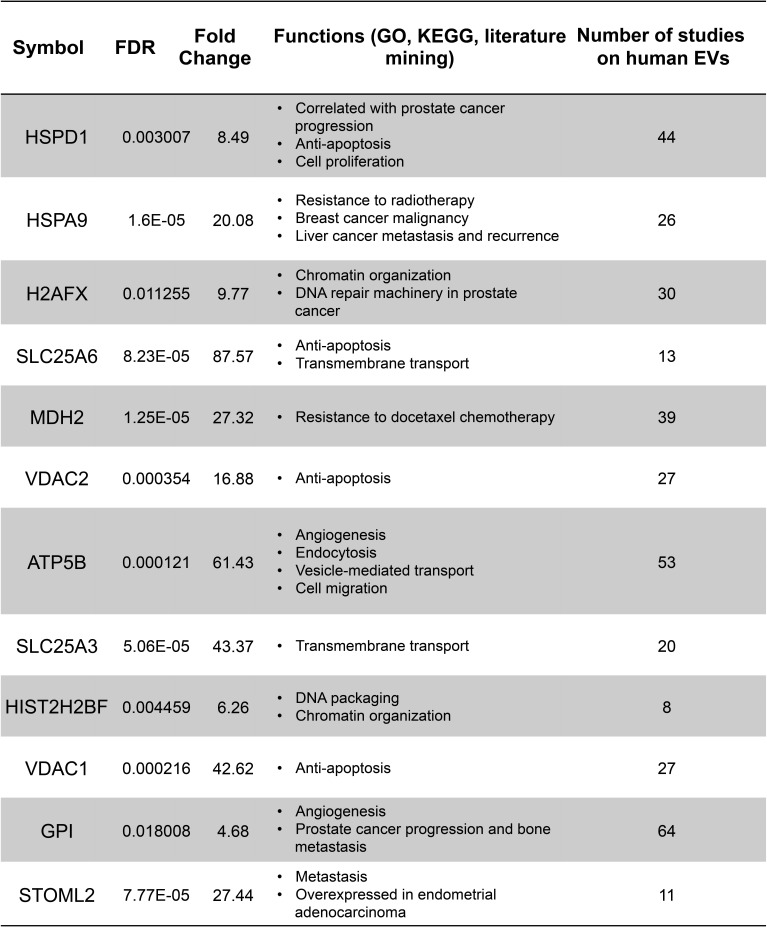
Proteins highly abundant in large EVs are associated with cancer progression Proteins with >4 fold enrichment in large EVs were functionally annotated by using the GO, KEGG and iHOP literature mining softwares to identify the association with cancer progression. The column on the right indicates the number of studies, obtained from the EVpedia database, in which these proteins were detected.

We also identified “unique proteins” in both large and nano-sized EVs using specific selection criteria (see Methods) (Figure 2B). Proteins uniquely identified in nano-sized EVs, similarly to the differentially expressed, were mainly involved in adhesion, motility, and response to hypoxia, functions frequently attributed to exosome proteins [32], whereas in large EVs metabolic enzymes were also predominant among the unique proteins. Notably, glutaminase (GLS), a cytoplasmic enzyme that converts glutamine to glutamate, was the most abundant protein uniquely identified in large EVs (Figure 2B). Further analysis demonstrated that several proteins significantly enriched (>4 fold) in large EVs and identified in the EV data repository EVpedia [32] were functionally categorized as playing a role in cell migration, resistance to docetaxel, angiogenesis, prostate cancer progression and bone metastasis, and other processes associated with tumor progression (Figure 3). These observations indicate that the two EV populations contain different cargo, and this might be a reflection of differences in function.

### Functional analysis of the proteins enriched in large EVs suggests an influence on cancer cell glutamine metabolism

**Figure 4:**
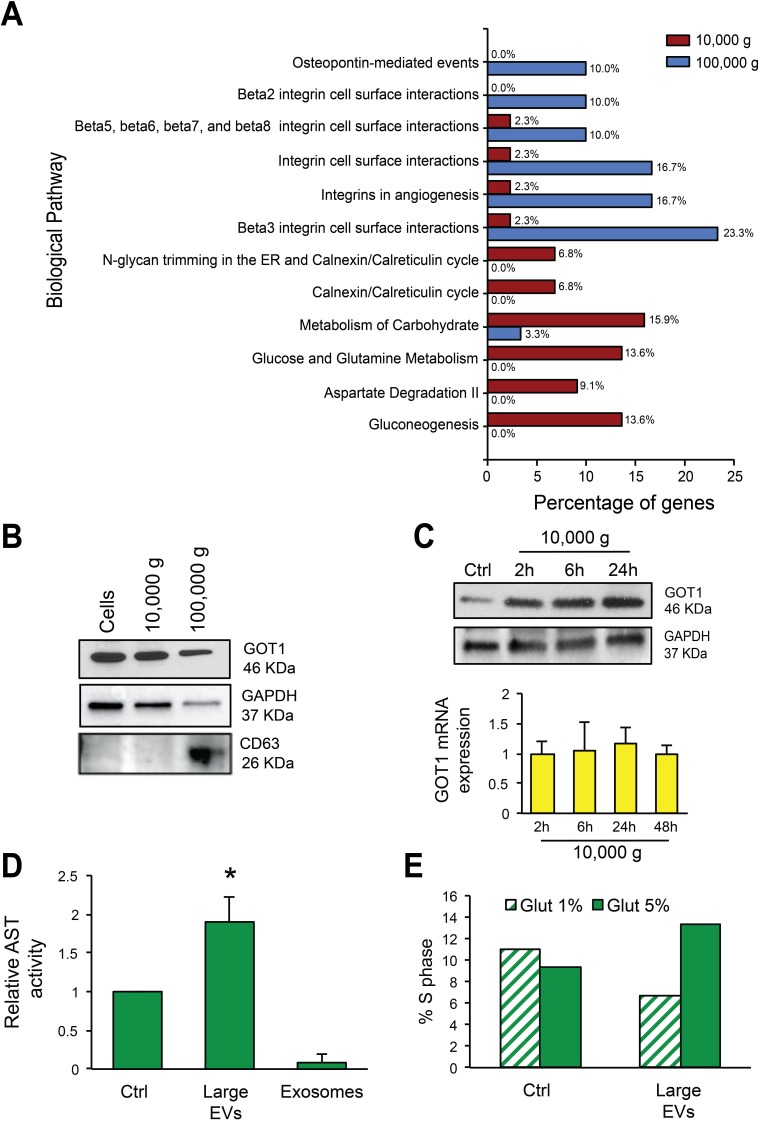
Large EVs are enriched in vesicular markers and alter glutamine metabolism in recipient cells (A) Functional analysis using FunRich software indicates the biological pathways overrepresented either in large (10,000 g) or nano-sized EVs (100,000 g). (B) Protein lysates from cells, large and nano-sized EVs were blotted with the indicated antibodies. CD63 was expressed specifically in nano-sized EVs, and GOT1 in large EVs. (C) Protein lysates from DU145 cells untreated or treated with large EVs for the indicated times, were blotted with GOT1 antibody. GAPDH was used as a loading control (top panel). GOT1 mRNA expression levels in DU145 cells untreated or treated with large EVs for the indicated times do not exhibit significant changes. The result is displayed as levels of GOT1 transcript after normalization to the housekeeping gene GAPDH in treated versus untreated cells at 2-48h (bottom panel). (D) GOT1 activity was measured in DU145 cells in 5% glutamine with or without treatment with large oncosomes (1.15 g/ml OptiPrep^TM^ density fractions) or exosomes (1.10 g/ml) (20μg/ml of protein lysate). The results from 3 experiments are displayed as relative AST activity in treated cells in comparison with the baseline activity of the enzyme (p=0.024). (E) Cell-cycle was analyzed by flow cytometry in DU145 cells treated with large oncosomes or vehicle in the presence of 1% or 5% glutamine for 24 hours.

Functional enrichment analysis using FunRich software (http://www.funrich.org) further indicated that proteins abundant in large EVs are involved in metabolism of carbohydrates (15.9%), glucose and glutamine metabolism (13.6%), aspartate degradation II (9.1%), and gluconeogenesis (13.6%), metabolic processes relevant to cancer [33] (Figure 4A). This result provided a rationale to determine whether large EVs were capable of inducing alterations in glutamine metabolism in recipient cells, a role that, to the best of our knowledge, has never been tested for any type of EV. Western blotting validated the enrichment of metabolic enzymes GOT1 and GAPDH in large EVs, whereas the tetraspanin CD63 was enriched in nano-sized EVs (Figure 4B). Increased expression of GOT1 was also observed in cancer cells exposed to large EVs as little as 2 h following exposure, suggesting that the protein might be transferred to recipient cells specifically via large EVs. GOT1 transcript levels were not altered by large EV treatment, thereby supporting protein, and not mRNA transfer to recipient cells (Figure 4C).

Because GOT1 catalyzes the reversible conversion from aspartate and α-ketoglutarate to glutamate, we used a functional assay that measures the production of glutamate per unit time as an indication of GOT activity in cancer cells exposed to large and nano-sized EVs [34]. Our results indicate a robust stimulation of the enzyme induced by large EVs, ultra-purified by buoyant density gradient as described in the following paragraph (Figure 4D). We also observed a robust increased activity of GOT in cells treated with large EVs but not vehicle, and cultured in 5% but not in 1% glutamine (Supplementary Figure 3A), suggesting that EVs stimulate tumor metabolic activities in the presence of non-rate limiting amounts of substrate. In line with this result, parallel experiments demonstrated an increased percentage of S-phase in cells treated with large EVs and cultured in 5% but not 1% glutamine (Figure 4E, Supplementary Figure 3B). These results indicate that large EVs can affect specific metabolic functions of cancer cells.

### Large EVs float to 1.10-1.15 g/ml in iodixanol centrifugation gradients

**Figure 5:**
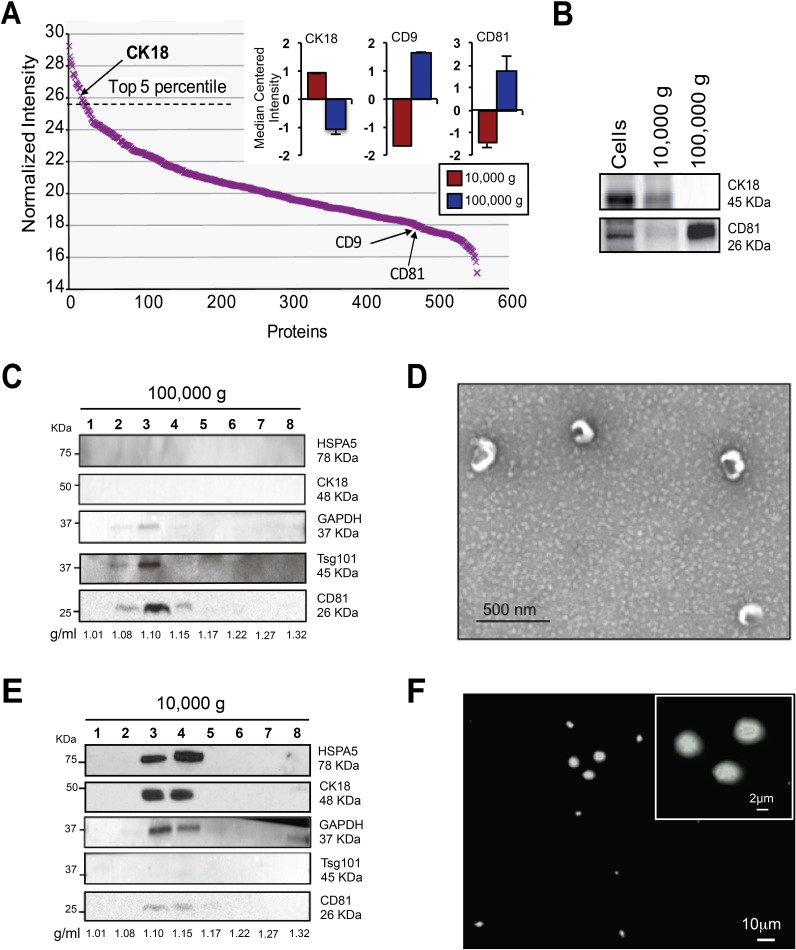
SILAC validation by OptiPrep^TM^ gradient, EM and IF (A) Rank plot of normalized ion intensities of all proteins identified in large EVs. CK18 is indicated as high abundant and CD9 and CD81 are indicated as low abundant proteins in large EVs. (B) Protein lysates from cells, large EVs and nano-sized EVs were blotted with the indicated antibodies. CD81 was expressed specifically in nano-sized EVs, and CK18 in large EVs. (C) Equal amounts of proteins (10 μg) from OptiPrep^TM^ fractions (1-8) of nano-sized EVs were blotted with the indicated antibodies. Exosome markers CD81 and Tsg101 were identified in fraction 3, corresponding to the buoyant density of 1.10 g/ml. (D) Magnified TEM detail of negative stained EVs corresponding to the buoyant density of 1.10 g/ml, showing cup-shaped vesicles. Scale bars, 500nm. (E) Equal amounts of proteins (10 μg) from OptiPrep^TM^ fractions (1-8) of large EVs were blotted with the indicated antibodies. Large EV enriched proteins (SILAC) such as CK18, GAPDH and HSPA5 were identified in fractions 3 and 4, corresponding to the buoyant density of 1.10-1.15 g/ml. (F) The 1.15 g/ml density fraction, labeled with DiO lipophilic dye, was imaged by IF. Scale bars, 10 μm and 2 μm (inset).

From the mass spectrometry analysis we found that CK18, a membrane-localized protein we previously used to decorate LO-like features *in situ* in human prostate cancer tissues [10], was highly abundant in large EVs (top 5^th^ percentile; Figure 5A). In contrast, CD9 and CD81 were expressed at negligible levels in large EVs (Figure 5A, inset; Supplementary Figure 2A). To further validate the SILAC findings, we performed immunoblotting of CK18, which was confirmed to be enriched in large EVs (10,000 × g) in comparison with nano-sized EVs (100,000 × g). In contrast, CD81 was over-represented in nano-sized EVs (Figure 5B).

To determine if the proteins identified using SILAC were associated with EVs, instead of protein clots or debris, and in order to determine the buoyant density of large and nano-sized EVs, we used iodixanol (OptiPrep^TM^), a medium that is less viscous than sucrose and therefore more likely to enhance the separation of EV populations with differing densities [15]. Large and nano-sized EV pellets, normalized to the same number of cells were separated by flotation in discontinuous 5-60% OptiPrep^TM^ density gradients following deposition of the EV material at the bottom of the tubes (fractionation by upward displacement). Western blot analysis of 10 μg of protein lysate obtained from the gradient fractions derived from the 100,000 × g pellets revealed a population of EVs expressing typical exosome markers, such as CD81 and tumor susceptibility gene 101 (Tsg101), which were detected at a buoyant density of 1.10 g/ml (Figure 5C). Transmission electron microscopy (TEM) of this fraction revealed a homogeneous population of round, cup-shaped vesicles with sizes ranging from 50 to 100 nm, consistent with exosome morphology [35] (Figure 5D). Western blot analysis of gradient fractions derived from the 10,000 × g pellets demonstrated that CK18, GAPDH and HSPA5, identified as potential large EV markers by mass spectrometry, floated at buoyant densities of 1.10 and 1.15 g/ml (Figure 5E). Levels of CD81 and Tsg101 in these fractions were negligible or undetectable. Microscopy of the 1.15 g/ml fraction, labeled with a fluorescent DiO lipophilic dye, revealed the presence of intact EVs, variable in size but larger than 1 μm, consistent with LO morphology as previously described [8, 10, 18] (Figure 5F).

### CK18 is a marker of large oncosomes and can be identified in the circulation and in tissues

Having validated enrichment of CK18 in large EVs and specifically in LO by western blotting (Figure 5B, E), we attempted to quantify LO shedding from shDIAPH3 cells by measuring the number of CK18 positive LO by FACS, using differentially sized beads (1-10 μm) to set the gates [10, 18]. We observed a 17-fold increase of events in the PE-positive channel when the EVs were stained with CK18 antibody in comparison with unstained vesicles (Supplementary Figure 4).

**Figure 6:**
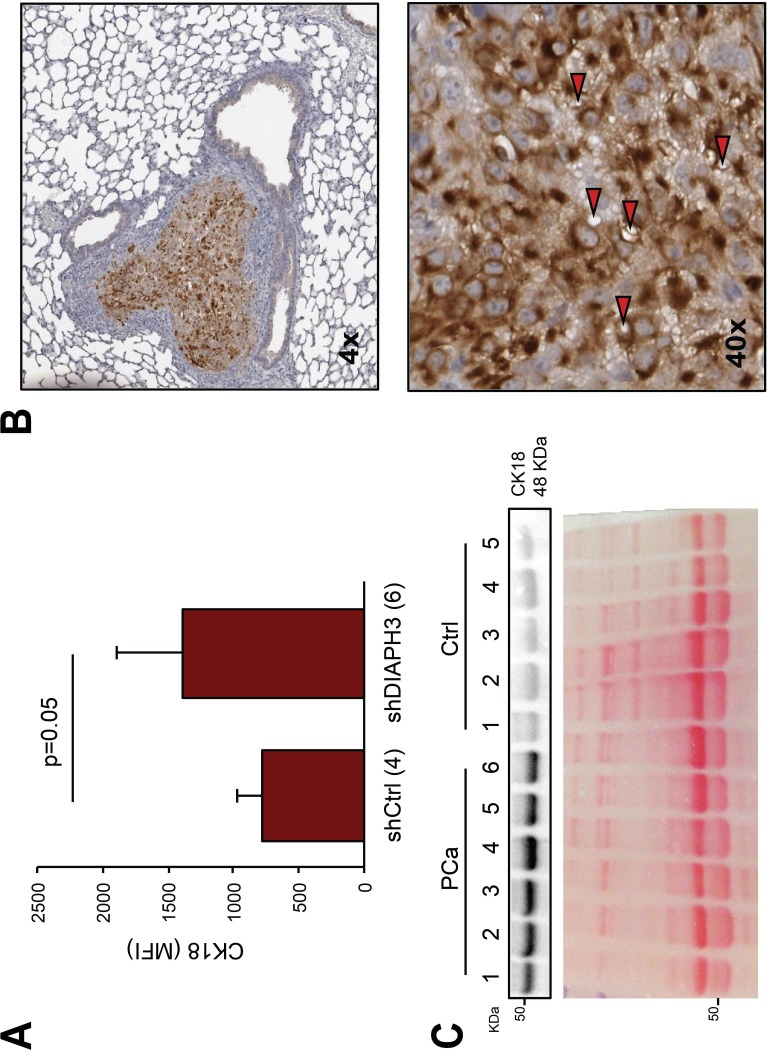
CK18 is a marker of large oncosomes *in vivo* (A) FACS analysis of CK18 positive EVs from the plasma of mice with lung metastasis injected with DIAPH3-silenced (n=6) or control (n=4) DU145 cells. The plot shows the mean fluorescent intensity (MFI) relative to CK18 positive EVs >1 μm. (B) Representative lung tissue section immunostained with CK18 (4X). The tumor is strongly positive for CK18, and LO features can be identified at higher magnification (40X). (C) CK18 western blotting of EVs isolated by ExoQuick^TM^ from the plasma of 6 patients with prostate cancer (PCa), and 5 healthy male subjects (Ctrl). Ponceau staining is displayed as a loading control.

We then took an analogous approach to quantitatively analyze circulating CK18 positive EVs >1 μm. We used plasma from a previously described mouse model in which shDIAPH3 DU145 cells, injected into the tail vein, formed a larger number of lung metastatic foci in comparison to control cells [10]. We observed a significant increase in the mean fluorescent intensity (MFI) of the CK18 signal in the plasma EVs of mice injected with shDIAPH3 DU145 cells in comparison to mice injected with control cells (Figure 6A). Importantly, the tumor tissue of the lung metastatic foci of the same animals expressed high levels of CK18, and exhibited LO-like features, strongly supporting a tumor origin for the large EVs detected in the plasma (Figure 6B).

Finally, as a first test for the potential use of CK18 as circulating marker in humans, total EVs were isolated from human plasma from prostate cancer patients using a commercial kit (ExoQuick^TM^) that does not distinguish between LO and exosomes [14, 16]. The rationale for using this kit is that it has the potential to be employed clinically to isolate EVs and other circulating tumor products. Levels of CK18 in ExoQuick^TM^ preparations from prostate cancer patients (n=6) were significantly higher than in healthy control subjects (n=5) (Figure 6C), suggesting that CK18 in EVs could potentially be used as a circulating cancer biomarker.

## DISCUSSION

This study reports the first comparative, large scale, quantitative analysis of the protein cargo of large and nano-sized EVs derived from a metastatic model of prostate cancer. We identified unique and differentially expressed proteins in the two EV populations. Glutamine and glucose metabolism emerged as biological pathways enriched in large versus nano-sized EVs. Large EVs with the appearance of particles described previously as LO could be purified using density gradient centrifugation [14, 15]. Furthermore, treatment with LO but not with exosomes altered aspartate transaminase activity in recipient cancer cells. Among the proteins enriched in LO, CK18 emerged as a marker for tumor-derived LO in tissues and in plasma.

Cancer cell-derived EVs can be heterogeneous in content, size, and density, and have been functionally implicated in the regulation of several processes during tumor growth and metastasis [5, 6, 36-39]. Despite the expectation that EVs derived from plasma membrane budding might carry different proteins than EVs derived from MVB, recent proteomic studies show a large overlap in proteins identified in different types of EVs from the same system [17, 40]. Therefore, the substantial overlap (69.9%) in proteins identified in this study in large and nano-sized EVs is corroborated by previous findings. However, the mass spectrometry findings reported here were obtained using a quantitative approach, and they indicate that levels of proteins identified in both EV types are quantitatively distinct. Importantly, although some of the proteins we identified in large EVs have been described as mitochondrial, the lack of cytochrome P450 argues against significant subcellular organelle contamination in our preparations [7]. Furthermore, membrane proteins constituted 30% of the proteins identified in both EV types, consistent with previous reports on EVs [24, 32]. Our findings that tetraspanins, growth factors (i.e. TGFβ1) and proteins related to cell adhesion are enriched in nano-sized EVs are consistent with previous reports on exosomes [41-43]. Similarly, cathepsin proteases, previously reported as EV cargo and involved in ECM degradation and remodeling, tumor progression, invasion and resistance to chemotherapeutic drugs, was identified in nano-sized EVs [29]. Additional studies are necessary to determine whether these cathepsin-containing EVs are tumor type-specific, and if they could be used as markers to purify distinct subtypes of EVs.

Our study newly demonstrates that we can purify large EV populations by floating medium-speed pellets on discontinuous density gradients. These particles localize to two discrete fractions, floating at 1.10-1.15 g/ml density, and shows morphology similar to LO described previously [8, 10, 18]. Notably, our density gradient of high-speed pellets allowed purification of pure populations of exosomes, floating at the original proposed density for this well-characterized EV population [14, 17, 44]. Further investigation and more granular gradient resolution will clarify whether LO have distinct physical properties in comparison with exosomes. On a functional point of view, while the relevance of exosomes to tumor progression is well established, data on LO are still limited. Our findings of a stronger association of LO cargo with aggressive cancer using GO, KEGG and iHOP literature mining softwares, along with the result that metabolism emerges as a LO-enriched biological process, suggest a distinct role for LO in tumor progression and indicate that these EV populations might play a selective metabolic function over other EV subclasses.

Altered glutamine metabolism in tumor cells, including a phenotype known as glutamine addiction, has been described in aggressive tumors such as glioma, melanoma and pancreas carcinoma [34, 45, 46]. We newly identified GOT1 as an enriched enzyme in LO and that LO can promote glutamine metabolism in recipient cancer cells by transferring the protein. However, this may not be the only mechanism. In fact, large EVs are also enriched in other proteins that affect glutamine metabolism, such as HSPA5 (GRP78), which was recently demonstrated to promote c-Myc-mediated glutamine flux and proliferation of cancer cells [31]. Additionally, HSPA5 expression has been correlated with tumor grade and metastatic events in triple negative breast carcinoma (TNBC) and is functionally relevant to clinical progression [47, 48]. Furthermore, GLS, a cytoplasmic enzyme that converts glutamine to glutamate is identified in large EVs as a unique protein. Alterations in glutamine and glucose metabolism are key aspects of the metabolic derangement in tumor cells and tumor-associated stroma [49], which can promote cancer cell proliferation. Additional implications of our findings are that LO may be involved in metabolic reprogramming of cells within the tumor microenvironment. This hypothesis will be the subject of future studies by our laboratory.

Our identification of CK18 as a protein significantly enriched in LO is in line with our previous identification of LO-like structures using CK18 IHC in human prostate cancer tissues. CK18 was recently detected in the circulation of patients with gastric and colorectal cancer [50, 51], and linked to a particular subtype of prostate cancer that aberrantly expresses p63, lacks the androgen receptor (AR) and harbors rearrangements of the ERG gene [52]. Expression of CK18 was also reported to be maintained in cancer cells after castration, in association with loss of AR and appearance of neuroendocrine markers, suggesting clinical utility for this luminal type cytokeratin [53]. However, this is the first report that identifies CK18 in plasma of patients with prostate cancer. This result is promising and suggests the possibility that this marker might be adapted for clinical assessment of disease status by employing quantitative, high-sensitivity detection methods (e.g., ELISA). Taken together, the results of this study support further investigation into the heterogeneous nature of tumor-derived EVs. They also suggest that the characterization of LO, as a distinct population from nano-sized exosomes, could result in the development of circulating diagnostic or prognostic signatures. Ongoing experiments in our laboratory are testing the feasibility of employing multiplex labeling of LO directly in body fluids.

## MATERIALS AND METHODS

### Cell culture

DU145 cells, expressing DIAPH3 hRNA or control vector, were cultured as previously described [10]. For SILAC labeling, DIAPH3-silenced DU145 cells were grown for at least six doublings in arginine- and lysine-depleted DMEM medium supplemented with 10% (v/v) dialyzed fetal bovine serum (Invitrogen, Grand Island, NY) and L-arginine (Arg0) and L-lysine (Lys0) or ^13^C_6_^15^N_4_-L-arginine (Arg10) and ^13^C_6_^15^N_2_-L-lysine (Lys8) (Cambridge Isotope Laboratories, Andover, MA), before undergoing isolation of large EVs and nano-sized EVs, respectively.

### Protein separation and digestion

Proteins were separated and digested as described [54]. SILAC-labeled proteins were mixed at a 1:1 (w/w) ratio, separated by 10% SDS-PAGE, and stained with Coomassie Brilliant Blue solution (Bio-Rad). Each lane was cut into ten gel slices of similar size and further cut into about 1 mm^3^ particles. Proteins were reduced by 10 mM dithiothreitol (DTT), alkylated by 55 mM iodoacetamide, and digested with mass spectrometry-grade trypsin (Promega) at 37°C for 16 h. Tryptic peptides were successively extracted with 100 μL of 5% acetic acid, 100 μL of 2.5% acetic acid and 50% acetonitrile, and 100 μL of 100% acetonitrile. Samples were dried in a SpeedVac concentrator (Thermo Scientific) and stored at −80°C until MS analysis.

### Liquid chromatography-tandem mass spectrometry

Tryptic peptides were redissolved with 20 μL 1.5% acetic acid and 5% acetonitrile. Samples (10 μL each) were analyzed by online C18 nanoflow reversed-phase HPLC (Eksigent nanoLC·2D™) connected to an LTQ Orbitrap XL mass spectrometer (Thermo Scientific) as described previously [55, 56]. Briefly, samples were loaded onto a 15 cm nanospray column (75 μm inner diameter, Magic C_18_ AQ, 3 μm particle size, 200Å pore size, Precision Capillary Columns) and separated at 300 nL/min with a 60-min gradient from 5 to 35% acetonitrile in 0.1% formic acid. MS data were acquired in a data-dependent manner selecting the fragmentation events based on the precursor abundance in the survey scan (350-1600 Th). The resolution of the survey scan was set at 30,000 at m/z 400 Th. Dynamic exclusion was 90 s. After each survey scan, up to ten collision-induced dissociation (CID) MS/MS spectra were acquired in the linear ion trap. The mass spectrometry proteomics data have been deposited to the ProteomeXchange Consortium (http://proteomecentral.proteomexchange.org) via the PRIDE partner repository [57] with the dataset identifier PXD000776.

### Protein identification and quantitation

The MS data were analyzed using MaxQuant (v1.3.0.5) [58]. Proteins were identified by searching MS/MS spectra against the UniProt database for Human (released on 09/11/2012, 84,680 entries) combined with 262 common contaminants by the Andromeda search engine [59], with second peptide identification enabled. Carbamidomethylation of cysteines was set as a fixed modification, oxidation of methionines and acetylation of the protein N-terminus as variable modifications. Trypsin allowing for cleavage N-terminal to proline was chosen as the enzyme specificity. A maximum of two missed cleavages were allowed. The minimum peptide length was specified to be seven amino acids. The maximal mass tolerance in MS mode was set to 20 ppm for first search and 6 ppm for main search, and in MS/MS mode 0.7 Da. The maximum false discovery rates (FDR) for peptide and protein identifications were specified as 0.01.

### Identification of differentially expressed proteins (DEPs) and functional enrichment analysis

Lists of DEPs (FDR < 0.05 and fold change ≥ 2) were identified by integrative hypothesis testing method as previously described [21, 60] using quantile normalized SILAC intensities [19]. Briefly, for each protein, we performed 1) two-tailed t-test and median ratio test; 2) computation of false discovery rates (FDRs) from the two tests using an empirical distribution of the null hypothesis (that the means of the genes are not different), which was obtained from random permutations of the samples; 3) combination of the two FDRs for the individual peptides using Stouffer's method [20]. In addition, to identify proteins uniquely detected in large or nano-sized EVs, we employed the following criteria: 1) being identified in at least one replicate with more than two sibling peptides and 2) being quantified in one single condition, not quantified in the other condition. The proteome of large and nano-sized EVs was analyzed by using functional enrichment analysis software FunRich (http://www.funrich.org).

### Isolation of large oncosomes and nano-sized EVs

Large EVs were purified by differential centrifugation (Beckman SW28 rotor) from conditioned medium or 300 μl of mouse platelet-poor plasma as previously described [10, 18]. Briefly, cells and debris were eliminated by centrifugation at 2,800 g for 10 min. The supernatant was then centrifuged at 10,000 g for 30 min to precipitate large EVs. For isolation of nano-sized EVs, the supernatant remaining after the 10,000 g spin was subjected to additional centrifugation at 100,000 g for 60 min. For discontinuous centrifugation gradient we used a modified version of a previously applied protocol [61]. Briefly 60%, 50%, 40%, 30%, 25%, 15%, 10% and 5% solutions were made by diluting a stock solution of OptiPrep™ (60% aqueous iodixanol from Sigma) in 0.25 M Sucrose/0.9 M NaCl/120 mM HEPES, pH 7.4. The 10,000 × g and 100,000 × g pellets were mixed in the bottom layer and the following solutions carefully layered. Centrifugation was performed at 100,000 × g for 3h and 50 min at 4 °C with a SW28 Beckman rotor. Eight individual fractions were collected, washed with PBS, and after centrifugation at 100,000 × g for 1 h at 4 °C, the pellet from each fraction suspended in either PBS or lysis buffer.

### FACS analysis of large EVs

Purified large EVs from conditioned media or mouse plasma samples (6 shDIAPH3 and 4 control) were washed in PBS, fixed and permeabilized with 0.5% Tween 20 and then stained with rabbit monoclonal CK18 (Abcam, 1:50). Samples were processed on a LSRII Flow Cytometer (BD) using 1, 2 and 10 μm bead standards [10, 18]. A minimum of 3000 events per experiment was recorded and the data analysis was performed with the FlowJo software (Treestar). The plot shows the mean fluorescent intensity (MFI). Statistical significance was calculated using a 2-tailed unpaired Student's t test.

### Isolation of EVs from human plasma

EVs were isolated from human plasma (in accordance with Ethics Board Approval Cert. H09-01010 obtained from the University of British Columbia, Canada) using precipitation solution ExoQuick™ (System biosciences) according to the manufacturer's manual with a few modifications. Briefly, 500 μl of plasma samples from prostate cancer patients and disease-free normal controls were diluted with 1X PBS (1:1). ExoQuick™ solution was added to the plasma, gently mixed and stored at 4° C overnight. The plasma samples were centrifuged at 1,500 × *g* for 30 min and the resulting exosome pellet was suspended in PBS. EVs pellet was stored at −80°C until further analysis.

### Fluorescence microscopy

Cells were stained with FITC-conjugated cholera toxin B (CTxB) subunit (Sigma) and imaged using an Axioplan 2 microscope (Zeiss), as previously described [8, 10, 18]. Alternatively, control or DIAPH3-silenced cells were imaged by a 20x objective on an Ultravox Spinning Disc Confocal microscope at the Boston Children's Hospital Intellectual and Developmental Disabilities Research Imaging Core facility (IDDRC, NIH-P30-HD-18655). The 25% iodixanol fraction, corresponding to the 1.15 g/ml fraction were stained with the lipophilic tracer DiO (Invitrogen) and imaged using a Leica TCS SP spectral confocal microscope.

### Transmission electron microscopy

EVs from the 15% iodixanol fraction 100,000 × g, corresponding to the 1.10 g/ml fraction were washed in HEPES, fixed with 1% glutaraldehyde and layered onto a mesh copper grids. Grids were then stained with the Negative Staining (NS) technique. Imaging was performed at an acceleration voltage by a transmission electron microscope JEM1200EX (JEOL, Japan) provided with a BioScan 600W digital camera (Gatan, USA).

### Immunoblot analysis

Cells, purified large EVs and nano-sized EVs were lysed and analyzed by western blotting using the following antibodies: rabbit monoclonal GOT1 (Sigma, 1:1000 dilution), rabbit polyclonal CD63, clone H-193 (Santa Cruz, 1:1000 dilution), rabbit polyclonal CK18 (Abcam, 1:1000 dilution); rabbit polyclonal CD81, clone M38 (Abcam, 1:1000 dilution), mouse monoclonal TSG101, clone C-2 (Santa Cruz, 1:500 dilution), rabbit monoclonal HSPA5 (Cell Signaling, 1:1000), and GAPDH (Cell Signaling 1:2000).

### Immunoblot and qRT-PCR analysis for GOT1 in cells exposed to EVs

DU145 cells, exposed to vehicle or large EVs for the indicated times, were blotted with GOT1 and GAPDH antibodies. mRNA from DU145 cells, exposed to vehicle or large EVs for the indicated times, was analyzed by qRT-PCR with commercially available specific primers for GOT1 and GAPDH (Hs.PT.58.45409452, and Hs.PT.39a22214836, respectively) (Integrated DNA Technologies).

### Aspartate Aminotransferase Activity Assay

Parental DU145 cells were treated with either 1.15 g/mL (large EVs) or 1.10 g/mL (exosomes) density fractions (20μg/ml of protein lysate) for 24 h in presence of 1% or 5% glutamine and then analyzed by using an Aspartate Aminotransferase Activity Assay kit following the manufacturer's instructions (Sigma). Statistical significance was calculated using a 2-tailed unpaired Student's t test among the relative biological replicates.

### Cell cycle analysis of DU145 cells exposed to large oncosomes

1×10^5^ DU145 cells were plated in 6 well plates overnight and then treated with large EVs or vehicle for 24 h, in presence of 1% or 5% glutamine. Cells were fixed, permeabilized, treated with RNAses (50μg/ml), and labeled with Propidium Iodide (5μg/ml). At least 10,000 events per experiment were recorded using a LSRII cytometer (BD Biosciences) and a BD FACSDiva software. Data analysis was performed with the FlowJo software (Treestar).

### Lung mouse tissue staining

Lung mouse tissues from animals injected with shDIAPH3 DU145 cells into the tail vein were immunostained with CK18 [9] and imaged by light microscopy.

## SUPPLEMENTARY MATERIAL, FIGURES AND MOVIE




