# Supplementary material for: Large oncosomes contain distinct protein cargo and represent a separate functional class of tumor-derived extracellular vesicles
Source: Oncotarget. 2015 Mar 14;6(13):11327–41. doi: 10.18632/oncotarget.3598 (PMC4484459; doi:10.18632/oncotarget.3598)
Supplement: Supplementary file 1 [file oncotarget-06-11327-s001.pdf]

Large oncosomes contain distinct protein cargo and represent a separate functional class of tumor-derived extracellular vesicles

Supplementary Material

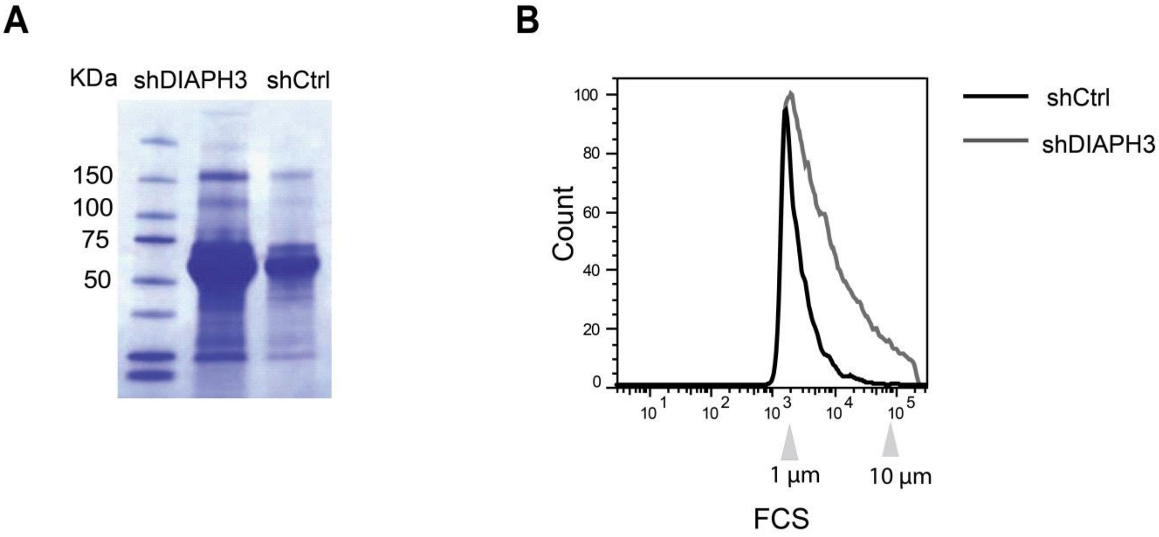

Figure S1: (A) Protein lysates from large EVs, isolated from the growth medium of DIAPH3-silenced or unsilenced control DU145 cells, were resolved by SDS-PAGE and stained with Coomassie Blue. Notice protein abundance is higher in EVs from DIAPH3-silenced cells. **(B)** Histogram of large EVs derived from DU145 cells stably transfected with either control or DIAPH3 shRNAs, showing increased EV shedding, compatible with large oncosomes, from the latter.

A. List of proteins enriched in large EVs.

Protein ID	Symbol	Large EVs/nano-sized EVs	FDR
P12236	SLC25A6	87.575	< 0.001
J3KPX7	PHB2	73.812	< 0.001
P06576	ATP5B	61.432	< 0.001
Q00325	SLC25A3	43.373	< 0.001
P21796	VDAC1	41.622	< 0.001
P00505	GOT2	29.161	< 0.001
Q9UJZ1	STOML2	27.436	< 0.001
P40926	MDH2	27.318	< 0.001
P38646	HSPA9	20.08	< 0.001
P45880	VDAC2	16.884	< 0.001
P05141	SLC25A5	15.667	0.001
P02751-15	FN1	10.702	0.003
P34897	SHMT2	9.967	0.003
P16104	H2AFX	9.772	0.011
P10809	HSPD1	8.491	0.003
Q13011	ECH1	8.138	0.008
P30048	PRDX3	7.905	0.008
Q02978	SLC25A11	7.786	0.008
P52272	HNRNPM	6.915	0.01
B4DR52	HIST2H2BF	6.262	0.004
Q9H3N1	TMX1	5.466	0.01
F8VTL3	MYH10	5.25	0.018
F5H7K4	NCEH1	5.208	0.01
P30084	ECHS1	4.924	0.01
Q969H8	C19orf10	4.899	0.021
P51571	SSR4	4.836	0.021
P23284	PPIB	4.683	0.021
P06744-2	GPI	4.677	0.018
Q15149	PLEC	4.613	0.024
P14314	PRKCSH	4.604	0.005
P20618	PSMB1	4.446	0.024
Q96AG4	LRRC59	4.409	0.025
P17174	GOT1	4.171	0.032
Q00839	HNRNPU	4.012	0.04
P04843	RPN1	4.007	0.033
P05783	CK18	3.977	0.003
P84103	SRSF3	3.94	0.013
P27797	CALR	3.896	0.031
P30101	PDIA3	3.89	0.038
P62805	HIST1H4A	3.865	0.014
P06748	P06748	3.839	0.038
P28074	PSMB5	3.718	0.03
P07237	P4HB	3.708	0.049
P13667	PDIA4	3.444	0.046
P49720	PSMB3	3.43	0.041
P42704	LRPPRC	3.414	0.048
P11021	HSPA5	3.359	0.014
P30040	ERP29	3.259	0.034
P35579	MYH9	3.256	0.01
F5H098	MDH1	3.217	0.006
E7EPA7	TKT	2.963	0.033
P07195	LDHB	2.851	0.019
P04406	GAPDH	2.777	0.017
B5MDF5	RAN	2.776	0.023
Q15084-2	PDIA6	2.528	0.041
P46782	RPS5	2.362	0.023
P07910	HNRNPC	2.006	0.027

B. List of proteins enriched in nano-sized EVs.

Protein ID	Symbol	Large EVs/nano-sized EVs	FDR
G8JLH6	CD9	10.133	0.01
A6NMH8	CD81	9.484	0.003
P11047	LAMC1	8.821	0.01
O94985	CLSTN1	8.374	0.003
P10253	P10253	7.527	0.013
Q14118	DAG1	6.995	0.016
P01137	TGFB1	6.973	0.016
P11166	SLC2A1	6.867	0.016
Q15758	SLC1A5	5.523	0.014
O75787	ATP6AP2	5.523	0.009
P10909-2	CLU	5.377	0.009
P08572	COL4A2	5.353	0.024
P62879	GNB2	5.346	0.011
P26006	ITGA3	5.326	0.012
J3KN08	MATN2	5.215	0.024
P19021-5	PAM	5.107	0.025
P27105	STOM	5.047	0.025
O00468	AGRN	4.904	0.05
P06756	ITGAV	4.748	0.026
Q08431	MFGE8	4.693	0.029
P18065	IGFBP2	4.692	0.012
P35052	GPC1	4.626	0.029
Q13641	TPBG	4.588	0.03
P31431	SDC4	4.55	0.032
P16070	CD44	4.465	0.046
P05362	ICAM1	4.449	0.034
Q9Y4K0	LOXL2	4.354	0.004
P62873	GNB1	4.26	0.038
I3NI00	BSG	4.249	0.001
O00159	MYO1C	4.205	0.04
B7Z5Z2	RRAS2	4.198	0.041
J3KNY4	CTSA	4.151	0.003
P07996	THBS1	4.123	0.018
Q9NS15	LTBP3	4.068	0.03
Q92820	GGH	4.043	0.044
P00749	PLAU	3.955	0.047
O43854	EDIL3	3.886	0.002
P21589	NT5E	3.87	0.048
P05067	APP	3.853	0.002
P16035	TIMP2	3.508	0.023
Q9UBV8	PEF1	3.354	0.01
P07339	CTSD	3.12	0.037
F5H3A1	ATP1A1	3.106	0.026
G8JLA8	TGFBI	3.008	0.045

Figure S2: Ratio of significant differentially expressed protein (DEPs) in large and nano-sized EVs **(A)** and vice-versa **(B)**. FDR <0.01.

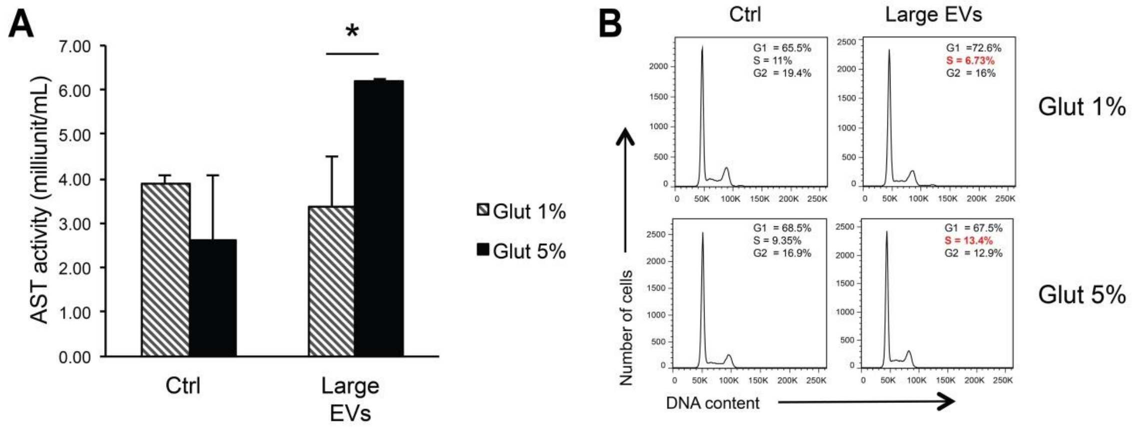

Figure S3: (A) The results from 2 experiments in DU145 cells treated with large oncosomes or vehicle are displayed as AST enzymatic activity (milliunit/mL), in cells cultured in 5% or 1% glutamine to highlight the enzymatic activity dynamically (p=0.023). **(B)** Cell-cycle analysis of DU145 cells treated with large oncosomes or vehicle in presence of 1% or 5% glutamine for 24 hours. After treatment, the cells were incubated with propidium iodide for DNA content-based evaluation of cell-cycle phase distribution. The percentage of cells in S-phase was increased by treatment with large oncosomes in 5% (13.4%) in comparison with 1% glutamine (6.73%). See also Fig 4.

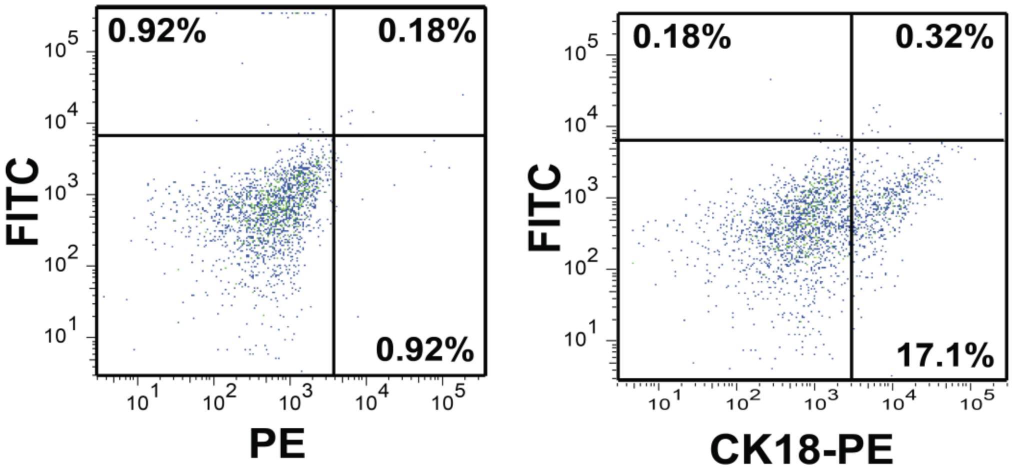

Figure S4: DIAPH3-silenced DU145 cell-derived large EVs unstained (left panel), and stained with CK18 (right panel) were analyzed by FACS.
